# Supplementary material for: Impact of Climate Change on the Distribution of Three Rare Salamanders (Liua shihi, Pseudohynobius jinfo, and Tylototriton wenxianensis) in Chongqing, China, and Their Conservation Implications
Source: Animals (Basel). 2024 Feb 21;14(5):672. doi: 10.3390/ani14050672 (PMC10931183; doi:10.3390/ani14050672)
Supplement: Supplementary file 1 [file animals-14-00672-s001.zip › Figure S1-2.pdf]

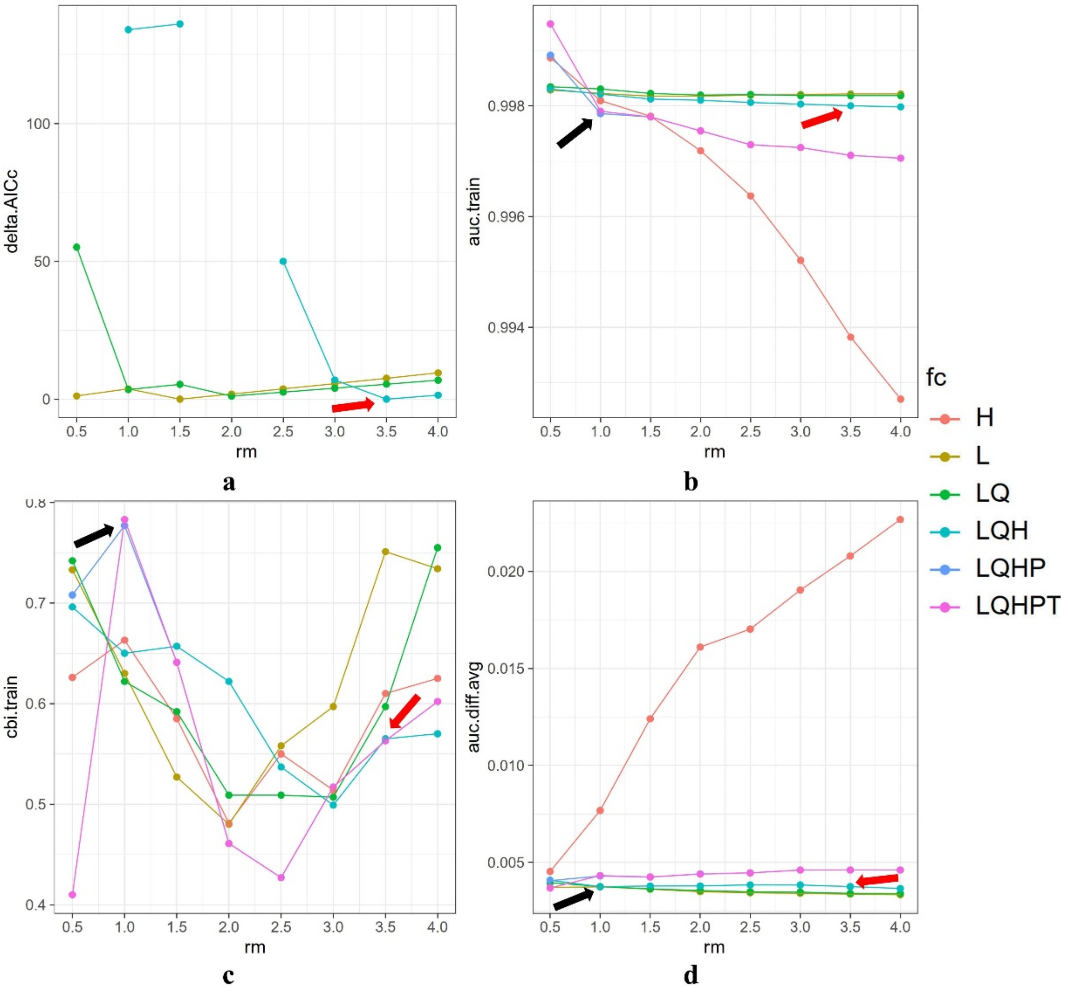

Figure S1. Optimization results for MaxEnt mode of *Pseudohynobius jinfo* under different parameter settings, (**a**) delta.AICc, (**b**) AUC.train, (**c**) CBI.train and (**d**) AUC.diff.

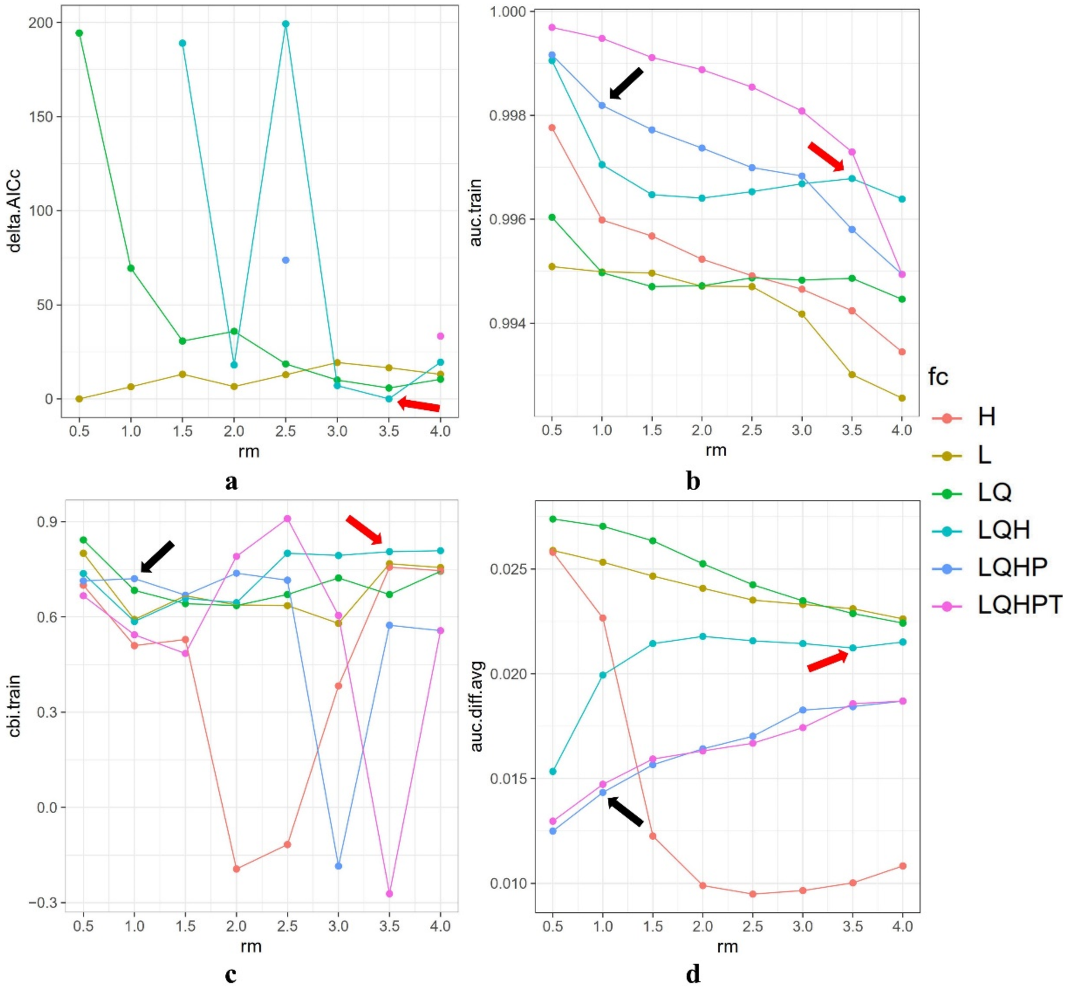

Figure S2. Optimization results for MaxEnt mode of *Tylototriton wenxianensis* under different parameter settings, (**a**) delta.AICc, (**b**) AUC.train, (**c**) CBI.train and (**d**) AUC.diff.
