# Supplementary material for: Impact of Climate Change on the Distribution of Three Rare Salamanders (Liua shihi, Pseudohynobius jinfo, and Tylototriton wenxianensis) in Chongqing, China, and Their Conservation Implications
Source: Animals (Basel). 2024 Feb 21;14(5):672. doi: 10.3390/ani14050672 (PMC10931183; doi:10.3390/ani14050672)
Supplement: Supplementary file 1 [file animals-14-00672-s001.zip › Table S1-3.pdf]

Table S1. Environmental variables for *Liua shihi* distribution prediction.

Environment variable	Variable type	Percent contribution (%)
DRO	Distance to the nearest road/m	74.7
Bio11	Mean Temperature of Coldest Quarter/°C	13.6
Bio3	Isothermality [(BIO2/BIO7)×100]	5.5
Bio13	Precipitation of Wettest Month/mm	2.2
EL	Elevation/m	2.1
DRE	Distance to the nearest residential area/m	1
AS	Aspect	0.8
Bio4	Temperature Seasonality	0.1

Table S2. Environmental variables for *Pseudohynobius jinfo* distribution prediction.

Environment variable	Variable type	Percent contribution (%)
Bio8	Mean Temperature of Wettest Quarter/°C	61.7
DRO	Distance to the nearest road/m	21.2
Bio2	Mean Diurnal Range/°C	15.1
Bio5	Max Temperature of Warmest Month/°C	1.1
DW	Distance to the nearest surface water/m	0.7
Bio10	Mean Temperature of Warmest Quarter/°C	0.2
EL	Elevation/m	0
Bio6	Min Temperature of Coldest Month/°C	0

Table S3. Environmental variables for *Tylototriton wenxianensis* distribution prediction.

Environment variable	Variable type	Percent contribution (%)
DW	Distance to the nearest surface water/m	58.2
DRO	Distance to the nearest road/m	30.5
EL	Elevation/m	4.5
DRE	Distance to the nearest residential area/m	2.4
bio11	Mean Temperature of Coldest Quarter/°C	2.1
bio2	Mean Diurnal Range/°C	1.2
NDVI	Normalized Difference Vegetation Index	1.1
bio4	Temperature Seasonality	0
