# Supplementary material for: Impact of Climate Change on the Distribution of Three Rare Salamanders (Liua shihi, Pseudohynobius jinfo, and Tylototriton wenxianensis) in Chongqing, China, and Their Conservation Implications
Source: Animals (Basel). 2024 Feb 21;14(5):672. doi: 10.3390/ani14050672 (PMC10931183; doi:10.3390/ani14050672)
Supplement: Supplementary file 1 [file animals-14-00672-s001.zip › Table S7-12.pdf]

Table S7. Suitable habitat area and proportion of the *Liua shihi* in Chongqing during different periods.

	Suitable area		Unsuitable area	
	Area (km^2)	Proportion (%)	Area (km^2)	Proportion (%)
Current	8007.40	9.72%	74392.60	90.28%
2050	10331.51	12.54%	72068.49	87.46%
2070	9869.08	11.98%	72530.92	88.02%
2090	7249.48	8.80%	75150.52	0.91

Table S8. Suitable habitat area and proportion of the *Pseudohynobius jinfo* in Chongqing during different periods.

	Suitable area		Unsuitable area	
	Area (km^2)	Proportion (%)	Area (km^2)	Proportion (%)
Current	891.76	1.08%	81508.24	98.92%
2050	254.72	0.31%	82145.28	99.69%
2070	164.44	0.20%	82235.56	99.80%
2090	58.97	0.07%	82341.03	99.93%

Table S9. Suitable habitat area and proportion of the *Tylototriton wenxianensis* in Chongqing during different periods.

	Suitable area		Unsuitable area	
	Area (km^2)	Proportion (%)	Area (km^2)	Proportion (%)
Current	671.48	0.81%	81728.52	99.19%
2050	307.00	0.37%	82093.00	99.63%
2070	169.50	0.21%	82230.50	99.79%
2090	46.01	0.06%	82353.99	99.94%

Table S10. Changes in the suitable habitat area of *Liua shihi* in Chongqing during different periods (km^2).

	Expansion	Stability	Contraction	Unsuitable
Current-2050	2391.31	8115.77	33.49	71859.43
2050-2070	10.54	10028.83	478.25	71882.38
2070-2090	0.48	7380.54	2658.82	72360.16

Table S11. Changes in the suitable habitat area of *Pseudohynobius jinfo* in Chongqing during different periods (km^2).

	Expansion	Stability	Contraction	Unsuitable
Current-2050	7.16	245.26	639.14	81508.43
2050-2070	0	162.98	89.44	82147.57
2070-2090	0	58.17	104.81	82237.02

Table S12. Changes in the suitable habitat area of *Tylototriton wenxianensis* in Chongqing during different periods (km^2).

	Expansion	Stability	Contraction	Unsuitable
Current-2050	0.34	310.38	367.49	81721.79
2050-2070	0	171.30	139.42	82089.28
2070-2090	0	46.46	124.85	82228.70
